# Eklavya—Do-It-Yourself Model: A Rolled Latex Sheet Conduit for Microsurgical Training

**DOI:** 10.1055/s-0045-1802555

**Published:** 2025-02-03

**Authors:** Girish Mirajkar, Uday Bhat, Amit Peswani, Sushrut Raut, Husain Adenwala, Sayali Samudre

**Affiliations:** 1 Department of Plastic Surgery, Topiwala National Medical College and BYL Nair Charitable Hospital, Mumbai Central, Mumbai, Maharashtra, India

**Keywords:** microsurgery training, education, micro-anastomosis, microsurgical model

## Abstract

**Objective:**

To introduce a simple and innovative low-fidelity microsurgical model using daily articles available in any plastic surgery operation theatre and to determine its quality, ease of use and cost-effectiveness.

**Materials and Methods:**

The model is essentially made by rolling a loose disposable glove upon a K-wire to create a true micro-vessel (< 2 mm). Adjustment in the size and thickness of the conduit can be made. Model was assessed by data obtained from subjective questionnaire to 29 experienced microsurgeons with more than 5 years of independent microsurgical practice. This experience of our model was compared with their experience with other material and animal tissue.

**Results:**

The chicken femoral is the best compared to rat model overall, but rolled latex is as good or second best as far as some of the features assessed.

**Conclusion:**

“Eklavya” microsurgical model provides a valuable alternative to traditional animal models, allowing trainees to practice and hone their skills without the ethical and financial concerns associated with live animal use.

## Introduction

Microsurgery is an essential component of plastic and reconstructive surgery training. Spaced interval repetition is the key to refine microsurgical skills. 1 Over the past 50 years, various training models have been developed. Historically, animal models have been a mainstay, but maintaining a wet laboratory involves multiple challenges, namely, cost, animal storage, tissue disposal, and human resources. Additional concerns about newly emerging zoonoses are growing. 2 With ethical treatment of animals and the high cost of maintenance, a need for a suitable alternative is felt.

This need has led to making models out of natural materials (leaves 3 ) and artificial materials, both commercially available and “do-it-yourself” (DIY) models made by learners themselves. The characteristics of an ideal microsurgical training model are the following:

- Real tissue-like feel.
- Ability to identify anticipated problems (e.g., anastomosis leak, bleeding, spasm, etc.).
- Incorporation of microscope handling, instruments, and knotting skills.
- Ability to train at routine workplace (outside animal laboratories).
- No ethical concerns.
- Easy availability.
- Easy reproducibility.
- Ability for objective skill evaluation.
- Cost-effectiveness.

However, none of the articles in the DIY category fulfilled all the above criteria. This article attempts to introduce a versatile DIY model using everyday material in the plastic surgery operating theater that fulfils nearly all the criteria. This model is very economical to make and is easily reproducible, helping young plastic surgeons hone their microsurgical skills in an objective manner. The purpose of this study is to describe the procedure of making the model. We also compare the usefulness of the described model to the other established microsurgical models.

## Materials and Methods

### Making the Model

The materials required for making are the following:

- Disposable examination gloves.
- Kirschner's wire (K-wire).
- Stationery glue.
- Scissors.

### Method



**Video 1** Video demonstrating the making of 'Eklavya' microsurgical model, its use and patency test.

**Fig. 1:**
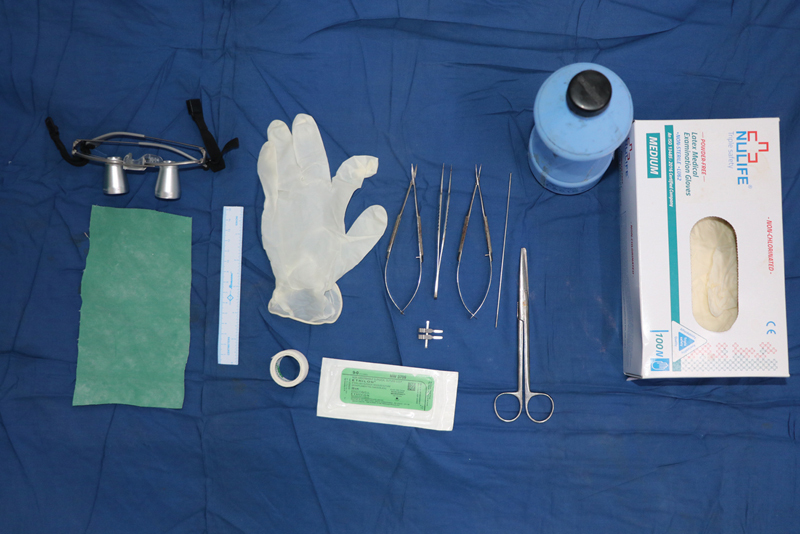
Materials required for making the rolled latex sheet, namely, latex examination glove, K-wire of desired size, stationery glue, and scissors. Remaining items for microsurgery practice.

**Fig. 2:**
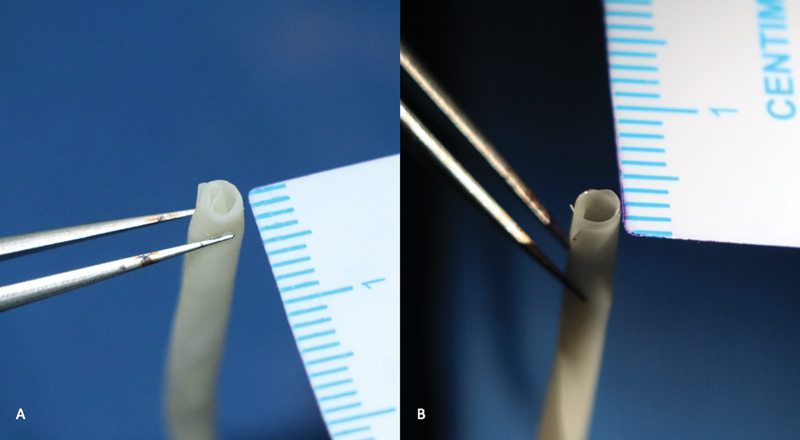
( **A** ) “Arterial vessel'” conduit having four rolls and ( **B** ) “venous vessel” conduit having two rolls.

- A disposable latex examination glove is used, and the finger of the glove is cut at both ends to create a cylinder.
- This cylinder is then cut open to form a rectangular flat sheet of latex measuring approximately 5 × 5 cm.
- The conduit is created by applying a thin layer of stationery glue to the latex sheet and rolling it over the K-wire ( Fig. 1 ).
- The thickness of the resultant “vessel” is determined by the number of rolls of latex, and its lumen diameter is determined by the K-wire of desired size (1, 1.2, 1.5, or 2 mm) as in shown in Fig. 2 (method elucidated in Video 1 , available in the online version only).
- The model can be adjusted according to the trainee's needs, for example, arterial (3–4 rolls) or venous (1–2 rolls).
- After creating the desired rolls, the excess latex sheet is discarded.

### Microsuturing Practice

On a table of comfortable height, a soft, stable wooden block may be kept for elevation. On this, the latex sheet conduit is placed and secured with stapler pins or microporous tape on its either side. A suitable background of medical soft rubber sheet may be used. The conduit can be cut in the center or an “arteriotomy/venotomy” may be created as required. We recommend the use of nonsterile practice polyamide sutures (8–0 or 9–0). If these are unavailable, then we suggest making microsutures with human hair swaged in a 30-gauge insulin syringe needle, as described by Al Azrak and Ozawa. 4 However, this process is tedious.

### End-to-End Anastomosis

**Fig. 3:**
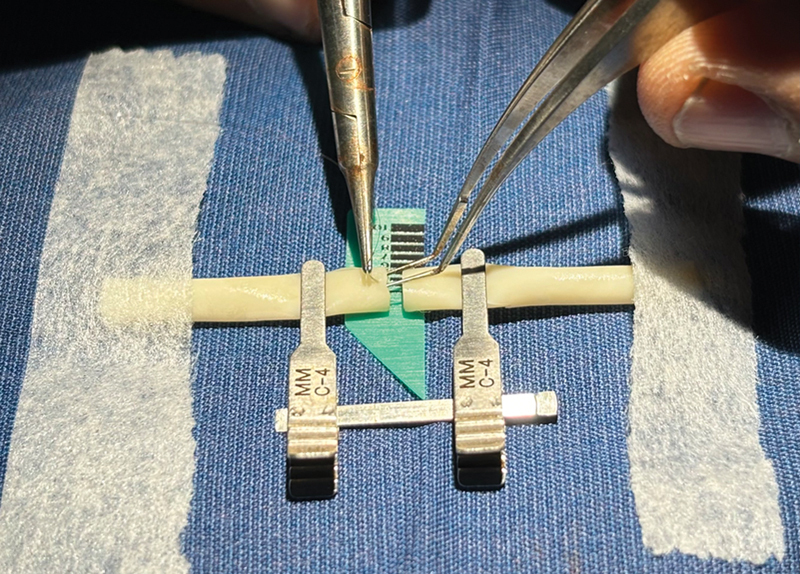
End-to-end anastomosis. The vessels are secured with microporous tapes and the business ends are held in place by vascular clamps for end-to-end anastomosis.

The “vessel” is perpendicularly cut in the center. The two segments are secured with microporous tapes on either ends and the business ends are held in place by vascular clamps for end-to-end anastomosis ( Fig. 3 ).

### End-to-Side Anastomosis

**Fig. 4:**
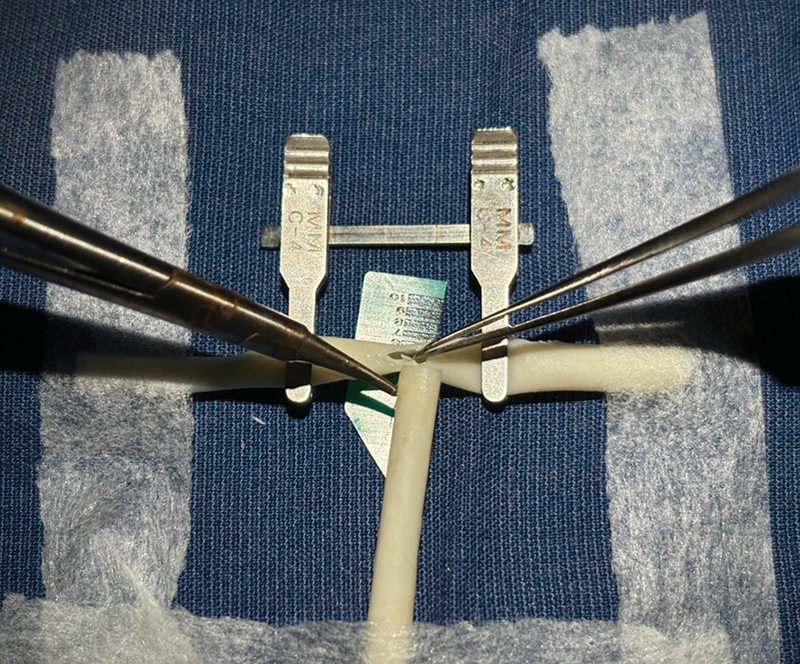
End-to-side repair. One conduit is juxtaposed at an angle to the other and their ends are secured with microporous tape. An “arteriotomy/venotomy” is made over the first conduit after application of vascular clamps.

Two conduits are used. One conduit is juxtaposed at an angle to the other and their ends are secured with microporous tape. An “arteriotomy/venotomy” is made over the first conduit after application of vascular clamps. Then an end-to side anastomosis is made ( Fig. 4 ).

### Performance of the Model with Respect to the Quality Testing of the Repair, Leak, and Patency

The repair performed on our model can be subjected to pretest and posttest Microsurgical Anastomosis Rating Scale-10 (MARS-10) grading 5 for assessment of the operator skills, tidiness, and patency of the anastomosis as performed on any other existing animal model. The MARS-10 grading is presented in Table 1 .

**Table 1:** Components of the 10-point Microsurgical Anastomosis Rating Scale (MARS10) grading system 5

Character assessed	Sutures		
**Score**	**0**	**1**	**2**
Parameter 1	Sutures not perpendicular to anastomosis Wrong bite sizes		Sutures perpendicular Equal, proper bites
**Score**	**0**	**1**	**2**
Parameter 2	Asymmetrical sutures Poor suture spacing		Symmetrical, parallel sutures Appropriate amount and suture spacing
**Character assessed**	**Knot quality**		
**Score**	**0**	**1**	**2**
Parameter 3	Cut ends too short/long Not square knot		Cut ends proper length Square knot
**Score**	**0**	**1**	**2**
Parameter 4	Inversion or eversion of vessel edges Stiches too loose or too tight		Appropriate tightness
**Character assessed**	**Anastomosis closure**		
**Score**	**0**	**1**	**2**
Parameter 5	Nonleakproof Obstructed anastomosis Back wall stich Sutures ends intraluminal		Leakproof, tight closure of anastomosis Nonobstructed anastomosis All sutures end extraluminal

**Fig. 5:**
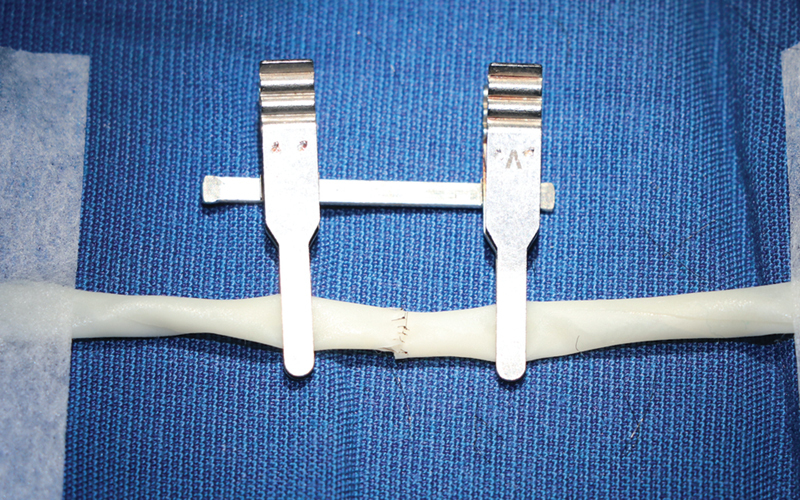
A completed end-to-end anastomosis on the model.

**Fig. 6:**
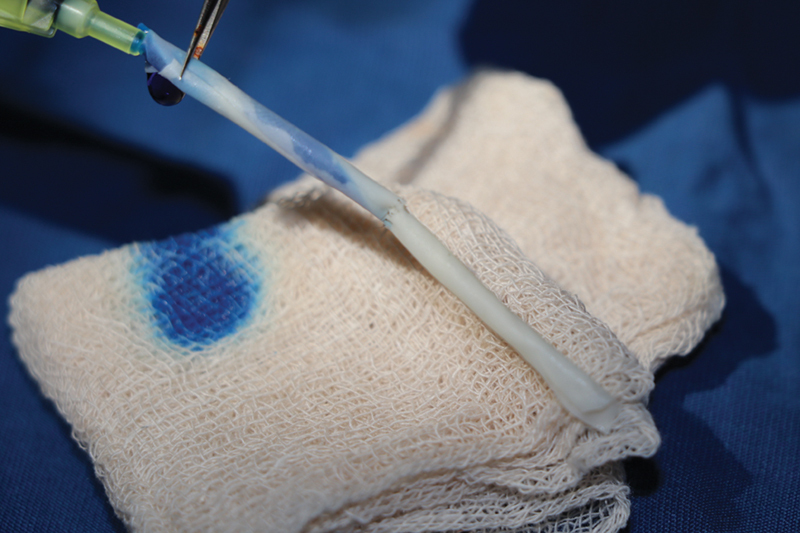
Patency test done by injecting diluted methylene blue from one end. This image shows that the anastomosis done is not patent as there is no free flow of methylene blue across it.

A leak is tested by injecting dilute methylene blue across the anastomosis site by having the other end clamped. The flow is tested by taking the clamp off and visualizing free flow of diluted dye. Another method of introducing dye is via an intravenous (IV) cannula connected to a used saline bottle with diluted methylene blue in it ( Figs. 5 and 6 ).

### Disposal after Use

The material after practice is treated as general waste. Sharps are disposed in appropriate containers.

### Assessment of Effectiveness of the Model

This model was assessed by 29 microsurgeons with experience in clinical microsurgery as well as familiarity with use of live animal models, chicken femoral arteries, and silicon conduits. Seventeen of these were senior plastic surgeons who had an experience of more than 5 years of independent clinical microsurgery and conduct microvascular training workshops routinely. This testing of the model was done in the dry laboratory of the authors' institute. A subjective assessment of the model was conducted under categories such as suture technique, quality control, and expenditure. 6 A written questionnaire ( Supplementary Material , available in the online version) was given to the microsurgeons after using the rolled conduit. The parameters were broadly classified as the following: ability to recreate anticipated events (e.g., leak, suturing through the posterior wall), suture technique, and quality control. The questions were answered on a scale from 1 to 5, where 1 = very poor, 2 = poor, 3 = fair, 4 = very good, and 5 = excellent.

Based on their experience on the rolled latex tube, silicone tube, chicken femoral artery, and live anesthetized rat (gold standard), the microsurgeons gave a scoring on the questionnaire. Data thus obtained were subjected to proportional analysis with a 95% confidence interval (using SPSS v.29 software). This experience of characteristics of the latex sheet conduit, chicken femoral artery, and silicon tube was compared with the gold standard, that is, experience of the live anesthetized rat.

## Results

The results of proportional analysis are as presented in Table 2 .

**Table 2:** Proportional analysis of the data obtained by a questionnaire of the rolled latex sheet conduit compared with a live rat model, chicken femoral artery, and silicon sheet

Features	Proportion with 95% CI (rat vs.)		
	Latex rolled sheet conduit	Chicken femoral artery	Silicon
Ability to recreate anticipated problems, e.g., leak, bleeding	86.21 (0.68–0.96)	62.07 (0.44–0.80)	68.97 (0.52–0.86)
Handling of the approximator clamps	93.10 (0.84–1.02)	93.10 (0.84–1.02)	79.31 (0.65–0.94)
Stitching and knotting	58.62 (0.41–0.77)	48.28 (0.31–0.66)	41.38 (0.23–0.59)
Vein interposition	62.07 (0.44–0.80)	51.72 (0.34–0.70)	51.72 (0.34–0.70)
Inspection of anastomosis	72.41 (0.56–0.89)	72.41 (0.56–0.89)	48.28 (0.31–0.66)
Patency test	75.86 (0.60–0.91)	75.86 (0.60–0.91)	72.41 (0.56–0.89)
Tightness of anastomosis	82.76 (0.69–0.97)	82.76 (0.69–0.97)	58.62 (0.41–0.77)
Recognition of Iatrogenic defects	55.17 (0.37–0.73)	48.28 (0.31–0.66)	48.28 (0.31–0.66)
Cost	96.55 (0.90–1.03)	100 (1–1)	89.66 (0.79–1.01)

Abbreviation: CI, confidence interval.

From Table 1 , following inferences can be drawn:

- Chicken femoral artery and rolled latex have equivalent ratings for the ability to identify anticipated problems feature.
- Chicken femoral is the best and silicon is the second best for stitching and knotting.
- For vein interposition, rolled latex is the best and chicken femoral is the second best.
- For handling of the approximator clamps, inspection of anastomosis, patency test, tightness of anastomosis, recognition of iatrogenic defects, and cost, we find that chicken femoral is the best and rolled latex is the second best.

In conclusion, the chicken femoral is the best compared with the rat model overall, but rolled latex is as good or second best as far as some of the features assessed.

## Discussion

We have classified microsurgical models into three types: animal, plant, and synthetic materials.

### Animal Models

They are further classified as live models (rat aorta or rat femoral vessels) and harvested vessel models (chicken femoral or wing vessels). 7 They mimic the human vessels closely. However, practice on animal tissues can be done only at animal laboratories that have logistical issues. Emerging zoonoses is an added hazard.

### Plant Models

Fresh leaf–based models 3 were initially described to practice fine skills due to their delicate nature. The prominent shortcoming of the model is its inability to have a rolled conduit.

### Synthetic Models

They may be classified into commercially available and the DIY models. Simple polyvinyl chloride tubes or silicon tubes are available in the market, but these lack tissue films. Practicerat (Sharpoint, Reading, PA, United States) 6 and polyvinyl alcohol hydrogel vascular model (KEZLEX) 8 have provided a near-realistic model that can be used multiple times. These models overcome the shortcomings of some of the animal models. They also provide an opportunity to have their anastomosis tested with a pulsatile flow of blood. However, their adoption is low due to high costs and limited availability.

The latex examination glove is an available to a trainee. Acland and Raja Sabapathy 9 described a model of a flat-cut latex glove stretched and fixed to a frame with adhesive tape. Although ingenious, it lacks a vessel like structure. Many authors have described preparations of a latex conduit, either by manufacturing single-layer microtubes with a K-wire by dipping in molten latex 10 (uniform conduits but no variability in the thickness) or by rolling them over an IV set tube 11 (large tubes, no variability in size). Some authors have described a model made of the solid rim of the ends of the glove 12 (no lumen). Hsieh and Chang 13 described a model whereby two parallel tubes are created with the use of a cut finger of the glove with longitudinally applied microporous tape. This model provides a single-layer tube of variable sizes as desired, which is one of its strongest points. Yet, none of these models have the ability for the microsurgical skills to be evaluated.

**Fig. 7:**
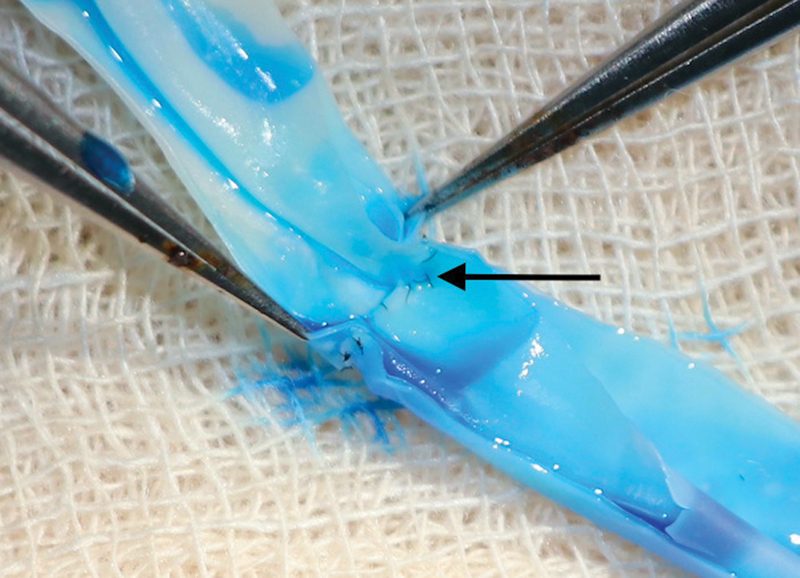
Back wall suture in the anastomosis shown in Fig. 6 after the conduit was laid open by cutting it longitudinally.

The proposed model is a multilayered latex conduit, created from the cut end of a disposable nonsterile glove finger within 5 minutes. The thickness of the vessel wall and thickness can be customized according to specific requirements, a feature not available in any existing models in the literature. According to feedback, the feel of the vessel closely resembles that of a human or animal model vessel despite the presence of glue between the layers. This model provides an opportunity to perform multiple end-to-end, end-to-side, and side-to-side anastomoses with “arterial” or “venous” vessels of varying diameters. The patency of the anastomosis can be evaluated using a 2-mL syringe with a 24-gauge cannula filled with diluted methylene blue. After anastomosis, the conduit can be cut open longitudinally to evaluate the quality of suturing using a MARS-10 grading as in Fig. 7 . This assessment can be conducted either in person or via telecommunication.

None of the existing DIY models offer the capability for microanastomosis quality assessment through the MARS-10 grading or leak testing. In this way, the proposed model addresses the gaps present in current models. Lack of an adventitia is of one of the shortcomings of this model.

## Conclusion

The rolled latex conduit model is an effective, easily reproducible, and cost-effective option for microsurgical training. It provides a valuable alternative to traditional animal models, allowing trainees to practice and hone their skills without the ethical and financial concerns associated with live animal use.
