# Supplementary material for: Eklavya—Do-It-Yourself Model: A Rolled Latex Sheet Conduit for Microsurgical Training
Source: Indian J Plast Surg. 2025 Feb 3;58(4):292–7. doi: 10.1055/s-0045-1802555 (PMC12396880; doi:10.1055/s-0045-1802555)
Supplement: Supplementary file 2 — Supplementary Material [file 10-1055-s-0045-1802555-s2492426.pdf]

Supplementary Material Questionnaire (tick the appropriate answer)

1. Name
2. Microsurgical experience
3. Tick next to the model you have used.
   a. Live anesthetized rat
   b. Chicken femoral artery
   c. Silicon tube
   d. Rolled latex sheet conduit
4. On a scale of

1. Very poor
2. Poor
3. Average
4. Very good
5. Excellent

Give a rating for each model for the following points

- Microdissection

	Live anesthetized rat	Chicken femoral artery	Silicon tube	Rolled latex sheet conduit
Handling of microscope				
Handling of instruments				
Microdissection				
Ability to recreate anticipated complications				
Feel of the vessel wall				

- Suture technique

	Animal model	Silicon tube	Latex tube
Handling of approximator clamps			
Stitching and knotting technique			
Vein interpositioning			

- Ability for quality control of suturing technique

	Animal model	Silicon tube	Latex tube
Inspection of anastomosis			
Patency			
Tightness of anastomosis			
Recognition of iatrogenous defect			

- Expenditure

	Animal model	Silicon tube	Latex tube
Ethical problems			
Stabling/storage			
Training in the OT			
Accessibility			
Interruption of exercise			
Cost			

 © 2025. The Author(s).
